# Rare coding variants of the adenosine A3 receptor are increased in autism: on the trail of the serotonin transporter regulome

**DOI:** 10.1186/2040-2392-4-28

**Published:** 2013-08-16

**Authors:** Nicholas G Campbell, Chong-Bin Zhu, Kathryn M Lindler, Brian L Yaspan, Emily Kistner-Griffin, William A Hewlett, Christopher G Tate, Randy D Blakely, James S Sutcliffe

**Affiliations:** 1 Department of Molecular Physiology & Biophysics and Vanderbilt Brain Institute, Vanderbilt University School of Medicine, Nashville, TN 37232-8548, USA; 2 Department of Pharmacology, Vanderbilt University School of Medicine, Nashville, TN 37232-8548, USA; 3 Department of Psychiatry, Vanderbilt University School of Medicine, Nashville, TN 37232-8548, USA; 4 Silvio O. Conte Center for Neuroscience Research, Vanderbilt University School of Medicine, Nashville, TN 37232-8548, USA; 5 Biostatistics and Epidemiology, Medical University of South Carolina, Charleston, SC 29425, USA; 6 MRC Laboratory of Molecular Biology, Hills Road, Cambridge CB2 0QH, UK

**Keywords:** Autism, Autism spectrum disorder, Serotonin, Serotonin transporter, Adenosine receptor, cGMP, DNA sequencing, Rare variant, Single nucleotide polymorphisms (SNPs), Allelic association

## Abstract

**Background:**

Rare genetic variation is an important class of autism spectrum disorder (ASD) risk factors and can implicate biological networks for investigation. Altered serotonin (5-HT) signaling has been implicated in ASD, and we and others have discovered multiple, rare, ASD-associated variants in the 5-HT transporter (SERT) gene leading to elevated 5-HT re-uptake and perturbed regulation. We hypothesized that loci encoding SERT regulators harbor variants that impact SERT function and/or regulation and therefore could contribute to ASD risk. The adenosine A3 receptor (A3AR) regulates SERT via protein kinase G (PKG) and other signaling pathways leading to enhanced SERT surface expression and catalytic activity.

**Methods:**

To test our hypothesis, we asked whether rare variants in the A3AR gene *(ADORA3)* were increased in ASD cases vs. controls. Discovery sequencing in a case-control sample and subsequent analysis of comparison exome sequence data were conducted. We evaluated the functional impact of two variants from the discovery sample on A3AR signaling and SERT activity.

**Results:**

Sequencing discovery showed an increase of rare coding variants in cases vs. controls (P=0.013). While comparison exome sequence data did not show a significant enrichment (P=0.071), combined analysis strengthened evidence for association (P=0.0025). Two variants discovered in ASD cases (Leu90Val and Val171Ile) lie in or near the ligand-binding pocket, and Leu90Val was enriched individually in cases (P=0.040). In vitro analysis of cells expressing Val90-A3AR revealed elevated basal cGMP levels compared with the wildtype receptor. Additionally, a specific A3AR agonist increased cGMP levels across the full time course studied in Val90-A3AR cells, compared to wildtype receptor. In Val90-A3AR/SERT co-transfections, agonist stimulation elevated SERT activity over the wildtype receptor with delayed 5-HT uptake activity recovery. In contrast, Ile171-A3AR was unable to support agonist stimulation of SERT. Although both Val90 and Ile171 were present in greater numbers in these ASD cases, segregation analysis in families showed incomplete penetrance, consistent with other rare ASD risk alleles.

**Conclusions:**

Our results validate the hypothesis that the SERT regulatory network harbors rare, functional variants that impact SERT activity and regulation in ASD, and encourages further investigation of this network for other variation that may impact ASD risk.

## Background

Serotonin (5-hydroxytryptamine (5-HT)) is a prominent monoamine neurotransmitter in the central and peripheral nervous systems, influencing mood, aggression, anxiety, impulsivity, and other behaviors. Serotonergic signaling has been implicated in multiple neuropsychiatric phenotypes, including major depression, obsessive-compulsive disorder (OCD), anxiety disorders, and autism spectrum disorder (ASD), among others (reviewed in ref. [1]). The presynaptic, antidepressant-sensitive 5-HT transporter (SERT; gene symbol: *SLC6A4*) is a critical regulator of 5-HT signaling by modulating synaptic 5-HT levels via presynaptic, Na^+^/Cl^-^ dependent re-uptake. Given the significance of SERT in regulating 5-HT function and its targeting by widely used medications, *SLC6A4* has been an attractive target for genetic studies in neuropsychiatric disorders. A significant focus of *SLC6A4* genetic studies is a common insertion/deletion polymorphism (5-HTTLPR) in the promoter region reported to impact *SLC6A4* gene expression [2]. Although some evidence supports association of 5-HTTLPR with psychiatric phenotypes including ASD, results overall are mixed [3-5], potentially influenced by the inherent difficulty of diagnosing behaviorally defined disorders and heterogeneity within and across cohorts examined. Additionally, single nucleotide variants (SNPs) within 5-HTTLPR and the promoter region are inherent confounds to many earlier studies [6-8].

The phenomenon of hyperserotonemia, or elevated whole blood or platelet 5-HT seen in approximately 35% of ASD cases, is the oldest ASD biomarker and is a highly heritable trait [9]. The presence of SERT on the platelet surface and its role in acquiring 5-HT from the blood provides a plausible biological mechanism for SERT involvement in hyperserotonemia [10-12]. Genetic association related to *SLC6A4* gene expression [13], as well as an interaction of *SLC6A4* with the gene encoding integrin β3 (*ITGB3*), which physically interacts with SERT, supports this idea [14-16].

Following observations of significant genetic linkage at 17q11.2 (harboring *SLC6A4*) in multiplex ASD families [17-19], we screened exons of *SLC6A4* specifically in families contributing to the observed linkage and found multiple, novel coding variants (Ile425Leu, Phe465Leu, and Leu550Val) and an elevated frequency of a previously documented coding variant (Gly56Ala) to a degree that profoundly deviated from expectations under Hardy-Weinberg equilibrium [17]. Further support for a role of these variants in ASD comes from studies reporting an Ile425Val variant that segregated in pedigrees harboring multiple psychiatric phenotypes, with Asperger syndrome (an ASD), OCD, and other anxiety disorders being the most prominent [20-22]. Functional characterization of these SERT variants revealed that each elevated 5-HT transport function, as well as altered protein kinase G (PKG) and p38 mitogen activated protein kinase (MAPK) regulation [23,24]. Our characterization of one of these variants (Gly56Ala) in knock-in transgenic mice revealed elevated 5-HT clearance and p38 MAPK-dependent transporter hyperphosphorylation *in vivo* accompanied by deficits in the three classical behavioral domains associated with ASD [25]. Collectively, these results suggest that altered 5-HT signaling, and SERT activity and/or regulation represents an important biological endpoint for the functional impact of genetic variation at other genes contributing to SERT regulation and ASD risk.

Modulation of synaptic 5-HT is a dynamic and tightly controlled process, subject to influence through multiple signaling pathways and interacting proteins that act on SERT (reviewed in [26]). Enhanced SERT activity can be achieved via PKG and p38 MAPK signaling pathways acting through trafficking-dependent and trafficking-independent (that is, functional modulation) mechanisms. A trigger for both of these uptake-enhancing pathways, and the focus of this paper, is activation of the A3 adenosine receptor (A3AR; gene symbol: *ADORA3*), a G-protein-coupled receptor (GPCR) that is expressed by 5-HT synthesizing neurons at synaptic terminals [27,28]. A3ARs physically interact and influence SERT [29] through a Gq-linked stimulation of guanyl cyclase (GC)-mediated cGMP synthesis. cGMP activation of PKG elevates SERT surface expression and, in parallel, a p38 MAPK-dependent elevation of surface resident SERT proteins [26,28,30]. Importantly, A3AR agonist stimulation of SERT is lost in A3AR knockout mice [30], providing evidence for the specificity of the current tools used to study receptor/transporter coupling.

Given a role of A3ARs in SERT regulation, we targeted *ADORA3* as a candidate locus to determine whether rare or common variants at this locus are correlated with ASD risk and/or with altered A3AR-mediated SERT function or regulation. To test for common allele effects on ASD risk, single nucleotide polymorphisms (SNPs) that index common haplotypes at *ADORA3* were assessed using family-based association methods. The alternative rare variant hypothesis was tested by Sanger sequencing of *ADORA3* exons in a sample of ASD probands and ethnically matched controls and followed by a replication analysis using data from whole exome sequencing of independent ASD cases and controls. Nonsynonymous variants identified as being increased in cases from these studies were evaluated functionally through heterologous expression of wildtype and variant A3ARs to test for changes in basal and agonist-activated modulation of SERT activity.

## Methods

### Subjects

#### Sample for allelic association analysis

The sample for analysis of common alleles in this study consisted of 958 combined simplex and multiplex ASD families (1,649 probands; 4,150 samples) recruited at Vanderbilt University, Tufts-New England Medical Center (including the Collaborative Linkage Study of Autism (CLSA)) [31], or obtained from the NIMH Genetics Repository (http://nimhgenetics.org). Samples from the NIMH Repository were submitted by one of three groups: the Autism Genetics Resource Exchange Consortium (AGRE), University of Iowa (CLSA) [31], or Stanford University [32]. All ASD probands were assessed with the Autism Diagnostic Interview (ADI) or its revision (ADI-R) [33] and most with the Autism Diagnostic Observation Schedule (ADOS) [34]. Affection status was assigned using a classification scheme employed by the Autism Genome Project [35] in which subjects were classified with either a ‘strict’ diagnosis if criteria for ‘autism’ were met on both the ADI-R and ADOS, or a more inclusive ‘spectrum’ (that is, ASD) diagnosis for subjects that met algorithm criteria for (1) ‘autism’ on the ADI-R alone; (2) ‘ASD’ [36] on both the ADI-R and ADOS; or (3) ‘autism’ on the ADOS alone. Other ‘unaffected’ family members were designated as ‘unknown’ for purposes of genetic analyses described below. Demographic information for this sample is shown in Additional file 1: Table S1. All studies were approved by the Vanderbilt Institutional Review Board and with the informed consent of participating families.

### Genotyping and analysis of common alleles at *ADORA3*

To test for association of common alleles at *ADORA3* in ASD, we selected SNPs to represent all common haplotypes (that is, ≥5%) across the transcriptional unit and flanking sequence taking into account linkage disequilibrium patterns. We used Haploview (http://www.broadinstitute.org/haploview) [37] to analyze CEPH (CEU; Caucasian) SNP data from the HapMap database (http://www.hapmap.org), given that the vast majority of the sample was of European ancestry. The Haploview implementation of Tagger identified tag-SNPs to capture common alleles across *ADORA3* at an *r*^2^ ≥0.8 and minor allele frequency (MAF) ≥5%. TaqMan™ allelic discrimination assays for four SNPs that span 14.4 kb were obtained from Applied Biosystems (ABI, Foster City, CA, USA) as Assays-on-Demand (AoD) or designed as Assays-by-Design.

PCR amplification was conducted in a 5-μL volume in accordance with manufacturer’s recommendations. In brief, cycling conditions included an initial denaturation at 95°C for 7 min, followed by 50 cycles of 92°C for 15 s and 60°C for 1 min. Post-PCR allelic discrimination was conducted using an ABI 7900HT genotypes ABI Sequence Detection System software. Genotypes were checked for completeness (≥98%) and conformity to expectations under Hardy Weinberg Equilibrium (HWE). Other quality control procedures included inter- and intra-plate replicates and checks for Mendelian inconsistencies using PEDCHECK [38]. Families containing Mendelian inconsistencies were identified and excluded from additional analysis. Family-based allelic association testing was used to evaluate transmission of alleles in the autism families being studied. Single marker analysis was conducted using the family based association test (FBAT) [39]. FBAT analysis was conducted under the additive model, and significance was determined using the empirical variance (−e) option, as this provides a more conservative estimate of association, given the presence of multiplex families in the dataset. Statistical power required to detect a meaningful association was determined by power calculations using the Genetic Power Calculator (http://pngu.mgh.harvard.edu/~purcell/gpc/) [40]. We assumed ASD as a discrete trait with a prevalence of 1/100 and a sample size of 1,000 trios, further assuming MAFs of 5%, 10%, and 30%, respectively, and a D´=0.8. Based on these calculations, we would have ≥80% power to detect odds ratios (ORs) of ≥1.59, ≥1.44, and ≥1.36 for (risk) allele frequencies of ≥5%, 10%, and 30%, respectively.

### Determination of ancestry from genotype data

To permit association analysis within the major European subset of the family-based sample and for subsequent matching of cases and controls (see below), ancestry was determined using STRUCTURE [41] and multidimensional scaling (MDS) in PLINK [42] (http://pngu.mgh.harvard.edu/~purcell/plink) to analyze genome-wide parental (founder) genotype data [35,43] from autism families. Genome-wide (GW) genotypes were derived from different platforms and in substantially different numbers. Many multiplex families were genotyped by the Autism Genome Project (AGP) in its Phase I linkage study using the Affymetrix 10 k SNP platform [44], and 10 k data were analyzed at that time using STRUCTURE. Other families had genotypes from Illumina 550 k [43] and/or 1 M SNP [35] arrays, and these were analyzed recently using MDS. Fortuitously, numerous families had both sets of genotypes and analyzed using both applications, and identical ancestry classifications provided confirmation that both STRUCTURE and MDS yielded consistent and robust assignments that also agreed with self-report information. A small number of families from the overall association sample (1) did not have GW genotypes, in which case self-report information determined classification or (2) had neither GW genotype data nor self-report information, in which case they were classified as being of ‘unknown’ ancestry.

### Discovery and analysis of rare variants at *ADORA3*

Initial screening for sequence variants at *ADORA3* utilized whole blood derived DNA samples from 185 unrelated (predominantly Caucasian) cases (94% Caucasian; 5% African-American; 1% Hispanic). Non-clinical comparison samples were drawn from reference collections and consisted of lymphoblastoid cell lines DNA from 305 subjects: (1) 96 samples from the ‘Caucasian’ subset of the Coriell Human Genome Diversity Panel (http://ccr.coriell.org/sections/Search/Panel_Detail.aspx?Ref=HD100CAU&PgId=202); (2) 192 samples from the Human Random Control (HRC) collection corresponding to subjects of European ancestry recruited in the UK and obtained from Sigma-Aldrich (St. Louis, MO, USA; http://www.sigmaaldrich.com/life-science/molecular-biology/pcr/human-genomic-dna.html); and (3) 24 subjects from the neurologically-normal NINDS/Coriell African-American panel (http://ccr.coriell.org/Sections/Search/Panel_Detail.aspx?Ref=NDPT111&PgId=202). Thus, the comparison samples were 92% Caucasian and 8% African-American. This discovery sample of cases and controls provides 80% power to detect rare risk variants of 0.1%, 0.5%, 1% allele frequencies with ORs of 16.2, 5.25, and 3.71, respectively. Our *a priori* expectation is that functional *ADORA3*-containing, SERT-altering functional variants would confer large genetic effects, and thus we are reasonably powered under this assumption. However, we would be significantly underpowered if hypothetical rare variants conferred ORs <2.

PCR amplifying primers (Additional file 2: Table S2) were designed using Primer3 (http://frodo.wi.mit.edu/primer3/), and all potential amplicons were subjected to a BLAST-Like Alignment Tool search to ensure no significant matches existed elsewhere in the genome. Amplicons were optimized using a gradient of annealing temperatures, followed by agarose gel electrophoresis to determine ideal annealing temperature. PCR was then performed on the case (185) and control (305) screening samples. PCR reactions contained 7.1 nM of amplifying primers, 10 μL of 2× Mastermix, and 12 ng of DNA in a final volume of 20 μL. *ADORA3* primers were designed for exons 1 to 2 and >500 bp of promoter sequence. PCR amplification conditions were 7 min at 95°C, 40 cycles of 15 s at 95°C, 12 s at the annealing temperature, and 60 s at 72°C, with a final extension at 72°C for 7 min. Following PCR, each product was examined by gel-electrophoresis, and once verified for expected size and specificity, excess primers and nucleotides were removed using Millipore 96 well filter plates. Samples were quantitated, subjected to Sanger sequencing, and data analyzed for variants using Sequencher v4.9 (Gene Codes Corporation, Ann Arbor, MI, USA). Specific variants were confirmed by independent PCR and sequencing, and for ASD cases with simultaneous determination of segregation by inclusion of other available family members, affected or unaffected.

Variants were assessed using multiple approaches. We noted whether or not they were previously documented in dbSNP or the 1000 Genomes project. *In silico* algorithms PolyPhen2 [45], SIFT [46], and SNAP [47] were used to provide a bioinformatic estimate of whether amino acid substitutions were likely to be ‘damaging’ or ‘not tolerated’ or benign. Cross-species conservation of the amino acid residue and surrounding sequence was also evaluated, along with available literature regarding structural features of the A3AR protein. Finally, to ask whether there might be evidence to support a global burden of rare (<1%) coding variants, the cohort allelic sums test (CAST) was used. CAST is a grouping method in which the number of individuals with one or more variants in a gene is compared between affected and unaffected individuals [48,49]. Thus, we compared differences in rare allele counts in matched cases and controls in a 2 × 2 contingency table, and a Fisher’s Exact test was performed.

An independent replication sample for comparison to variation identified in the discovery phase corresponded to ASD cases and controls from the NIMH Repository. Cases and controls were pair-matched for ancestry, and samples used for whole exome sequence analysis as recently described [50]. *SLC6A4* coding variants (described above) that affect SERT function and regulation are associated with not only ASD but also OCD, anxiety and mood disorders [17,20,21]. Therefore, case-control pairs were excluded if control subjects had any history of OCD or anxiety disorders. Sequence data at *ADORA3* for a total of 339 case-control pairs were examined for ‘functional’ (that is, missense, nonsense, consensus splice site, and read-through) variants. Details regarding quality control procedures and read depth for case and control samples are detailed in the Supplemental Material of Neale et al. [50].

### Functional studies

#### Constructs

A full-length cDNA encoding human SERT in the mammalian expression vector pcDNA3.1 (Invitrogen, Carlsbad, CA, USA) and a full-length cDNA encoding the human A3AR (myc-A3AR/pCMV) have been described previously [30]. *In vitro* mutagenesis of the A3AR cDNA clone to introduce Val90 and Ile171 variants were performed using the QuikChange mutagenesis kit (Stratagene, La Jolla, CA, USA). Variant constructs were later confirmed by direct sequencing of the entire open reading frame.

#### Cell culture and transfection

Chinese Hamster Ovary (CHO) cells were maintained at 37°C in Dulbecco’s modified Eagle’s medium containing 10% fetal bovine serum, 1% L-glutamine, 100 IU/mL penicillin, and 100 μg/mL streptomycin. Transfections were performed using the *Trans*IT-CHO Transfection reagent (Mirus, Madison, WI, USA). Quantities of 50 ng/well SERT and 20 ng/well of A3AR cDNAs were preincubated with Mirus reagent for 30 min at room temperature prior to adding to plated cells, seeded at 20,000 cells/well. Transfected cells were cultured for an additional 24-48 h after transfection and prior to assay of [^3^H]5-HT transport activities.

#### cGMP activity assays

The concentration of the second messenger cyclic guanosine monophosphate (cGMP) was measured using the CatchPoint cGMP fluorescent assay kit (Molecular Devices, Sunnyvale, CA, USA). In brief, CHO cells co-transfected with wildtype A3AR or A3AR coding variants and SERT cDNAs were plated at 50,000 cells/well. Cells were initially washed in a Krebs-Ringer bicarbonate (KRGB) prestimulation buffer containing 0.8 mM 3-isobutyl-1-methylxanthine (IBMX), an inhibitor of cGMP-phosphodiesterases. Following a 10 min incubation period, cGMP accumulation was measured in response to a 1 μM IB-MECA or vehicle treatment at various time points (0 min, 10 min, 20 min, 30 min, 40 min, 60 min) according to the manufacture’s protocol. The 1 μM IB-MECA concentration was selected on the basis of previous studies [28,30] that found optimal stimulation of A3ARs under those conditions. All values were normalized to 100% representing basal (that is, time = 0 min) wildtype (WT) cGMP levels. Statistical analyses comparing the effects of IB-MECA treatment on variant and wildtype A3ARs were performed with Prism (GraphPad, La Jolla, CA, USA) using two-way analysis of variance (ANOVA). We used a t-test to compare overall differences of IB-MECA treatment on variant and wildtype A3ARs for cGMP synthesis (and 5-HT below) over the full time course, area under the curve (AUC) was calculated with Prism using total cGMP production (or 5-HT uptake) for each 60 min time course experiment. Means and standard errors of the mean were calculated using each experiment. *P* values <0.05 were considered significant.

#### 5-HT transport assays

Assays measuring transport of [^3^H]5-HT were conducted as described previously [28,30]. Briefly, medium from CHO cells were removed and cells washed with Krebs-Ringer-Hepes (KRH) buffer containing 130 nM NaCl, 1.3 nM KCL, 2.2 mM CaCl_2_, 1.2 mM MgSO_4_, 1.2 mM KH_2_P0_4_, 1.8 g/L glucose, 10 mM HEPES, pH 7.4, 100 mM pargyline, and 100 mM ascorbic acid. Cells were incubated in triplicate at 37°C in KRH buffer containing 100 μM pargyline and 100 μM ascorbic acid, with and without the adenosine A3 selective agonist and adenosine analog IB-MECA. Following incubation with IB-MECA, a 10 min incubation with [^3^H]5-HT (20 nM) at 37°C was performed, followed by aspiration of buffer and three washes with ice-cold KRH buffer. Cells were then solubilzed with 0.5 mL Microscint-20, and [^3^H]5-HT accumulation was quantified using a TopCount plate scintillation counter (PerkinElmer Life and Analytical Sciences, Waltham, MA, USA). Specific 5-HT uptake was determined by subtracting the amount of [^3^H]5-HT accumulated in the presence of 10 μM paroxetine, a selective SERT inhibitor. A minimum of three independent replicates were performed for each experiment. Analyses comparing basal (time = 0 min) and IB-MECA-modified uptake for wildtype *vs*. variant A3AR/SERT co-transfections at individual time points and for the full time course was calculated and plotted using Prism as described above.

#### Molecular modeling to predict location of A3AR coding variants

The A3AR residue positions of Leu90 and Val171 were determined for the human adenosine A2a receptor (A2aAR) by amino acid sequence alignment and were found to correspond to Val84 and Val171, respectively. The relative positions and structural conformation of the corresponding A2aAR residues were determined using structural data obtained using an adenosine-bound, thermostabilized A2aAR (PDB ID:Y2DO) as a model. Images were prepared using PyMOL (DeLano Scientific Ltd).

## Results

### Family-based association analysis

To test whether common alleles at *ADORA3* contribute to ASD risk, we genotyped a sample consisting of 958 autism families using four SNPs representing common haplotypes that span the *ADORA3* locus. Primary FBAT analyses of genotype data were designed to test allelic transmissions along two axes of stratification: strict *vs*. spectrum diagnostic classifications and European *vs*. all ancestries, yielding four primary analyses. Analysis for each of the resulting four strata (strict-European and strict-all ancestries; spectrum-European and spectrum-all ancestries) showed no evidence to support common variant association at *ADORA3* using data for the four SNPs tested (Additional file 3: Table S3). Analysis of haplotypes (>5%) across the locus that were captured by these SNPs also showed no significant association, consistent with single marker analysis (data not shown).

### Sequence-based discovery of functional variation at *ADORA3*

To identify potentially functional, risk variants in *ADORA3*, we screened all exons and the promoter region in 185 unrelated ASD cases and 305 non-clinical comparison samples by direct Sanger sequencing. Comparison samples were selected to ethnically match case samples for Caucasian and African-American subjects, which represented the vast majority of the case screening panel (see Methods). Multiple synonymous and non-synonymous variants were identified and are documented in Table 1. Three novel variants, two non-synonymous (ns) and one synonymous, were detected in cases (Leu90Val, Val171Ile, Cys194) but were not previously documented in dbSNP or 1000 Genomes (1 kG) [51]. A novel Ala195Thr substitution was identified in a single control sample. Experimental validation of variants by sequencing independent PCR products included parents and other siblings (when applicable). No *de novo* variants were detected, and thus all were inherited in ASD families. Bioinformatic algorithms PolyPhen2 [45], SIFT [46], and SNAP [47] that predict the likelihood of functional effect of coding variants on protein function were applied to rare (<1%) ns-variants identified. Val171Ile (detected only in cases) was predicted to be ‘damaging’ or ‘not tolerated’, however, Leu90Val along with Ala195Thr were predicted to be benign substitutions. We note that subsequent to our discovery of novel Leu90Val and Ile171Val variants, but during subsequent functional and follow-up genetic experiments, both variants emerged from 1 kG sequence data and were thus deposited into dbSNP, as reflected in Table 1.

**Table 1:** **Sanger sequencing discovery of variation at *ADORA3***

				**Unrelated probands**		**Caucasian controls**		**African-American controls**	
**SNP ID**	**HGVS**^**a**^	**mRNA position**^**b **^**(NM_000677.3)**	**mRNA location**^**c**^	**Chromosomes**^**d**^	**MAF**^**e**^	**Chromosomes**^**d**^	**MAF**^**e**^	**Chromosomes**^**d**^	**MAF**^**e**^
rs10776728	c.108-13736A>T		-376	105/370	0.284	178/562	0.317	17/48	0.354
rs140137165	c.108-13682G>C		-322	0/370	0.000	0/562	0.000	2/48	0.042
	c.108-13643G>T		-283	0/370	0.000	1/562	0.002	0/48	0.000
rs10776727	c.108-13642G>T		-282	164/370	0.443	251/562	0.447	13/48	0.271
rs114241928	c.108-13599C>G		-239	0/370	0.000	0/562	0.000	2/48	0.042
	c.108-13532C>T		-172	1/370	0.003	0/562	0.000	0/48	0.000
	c.108-13371C>T		-11	0/370	0.000	2/562	0.004	0/48	0.000
	c.-724G>A	44	5'UTR	1/370	0.003	1/562	0.002	0/48	0.000
rs1544223	c.-581G>A	187	5'UTR	71/370	0.192	119/562	0.212	11/48	0.229
rs1544224	c.-564 T>C	204	5'UTR	73/370	0.197	130/562	0.231	11/48	0.229
	c.-563G>A	205	5'UTR	0/370	0.000	1/562	0.002	0/48	0.000
	c.-479A>G	289	5'UTR	0/370	0.000	1/562	0.002	0/48	0.000
rs41282522	c.-221G>C	547	5'UTR	52/370	0.141	81/562	0.144	6/48	0.125
rs41282520	c.-105A>C	663	5'UTR	1/370	0.003	4/562	0.007	0/48	0.000
	c.-85G>A	683	5'UTR	0/370	0.000	1/562	0.002	0/48	0.000
rs35789323	c.265C>T	1,032	Leu89Leu	1/370	0.003	0/562	0.000	0/48	0.000
rs77883500	c.268C>G	1,035	Leu90Val	3/370	0.008	0/562	0.000	0/48	0.000
rs76934313	c.345C>T	1,112	Thr115Thr	2/370	0.006	3/562	0.005	0/48	0.000
rs2789537	c.390C>T	1,157	Ala130Ala	6/370	0.016	5/562	0.009	1/48	0.021
rs139935750	c.511G>A	1,278	Val171Ile	3/370	0.008	0/562	0.000	0/48	0.000
	c.582C>T	1,349	Cys194Cys	1/370	0.003	1/562	0.002	0/48	0.000
rs143962803	c.583G>A	1,350	Ala195Thr	0/370	0.000	1/562	0.002	0/48	0.000
rs35511654	c.742A>C	1,509	Ile248Leu	54/370	0.146	84/562	0.158	6/48	0.125
rs2800889	c.797 T>A	1,564	Met266Lys	6/370	0.016	5/562	0.009	1/48	0.021
rs2229155	c.897 T>C	1,664	Ala299Ala	52/370	0.154	100/562	0.178	9/48	0.188
	c.*97C>A	1,821	3'UTR	0/370	0.000	1/562	0.002	0/48	0.000
rs923	c.*189C>T	1,913	3'UTR	51/370	0.149	100/562	0.178	9/48	0.188
rs1415793	c.*336C>T	2,060	3'UTR	51/370	0.149	100/562	0.178	9/48	0.188
rs75048140	c.*350A>G	2,074	3'UTR	2/370	0.005	0/562	0.000	0/48	0.000
rs1415792	c.*377A>G	2,101	3'UTR	50/370	0.146	100/562	0.178	9/48	0.188
	c.*409C>T	2,133	3'UTR	0/370	0.000	1/562	0.002	0/48	0.000
rs3393	c.*423G>A	2,147	3'UTR	123/370	0.435	258/562	0.459	9/48	0.188
rs3394	c.*441A>G	2,165	3'UTR	51/370	0.149	99/562	0.176	9/48	0.188

^a^Human Genome Variation Society (HGVS) recommended nomenclature. * Indicates substitution 3’ of termination codon.

^b^Indicates base-pair position within mRNA strucutre. RefSeq Accession number is given in parenthesis.

^c^Indicates location within mRNA structure (Exon). Negative (-) numbers indicate base pair position 5' to mRNA. Amino acid substitutions are shown relative to protein position.

^d^Indicates the number of minor allele counts over the total number of chromosomes sequenced.

^e^MAF indicates minor allele frequency.

**Figure 1:**
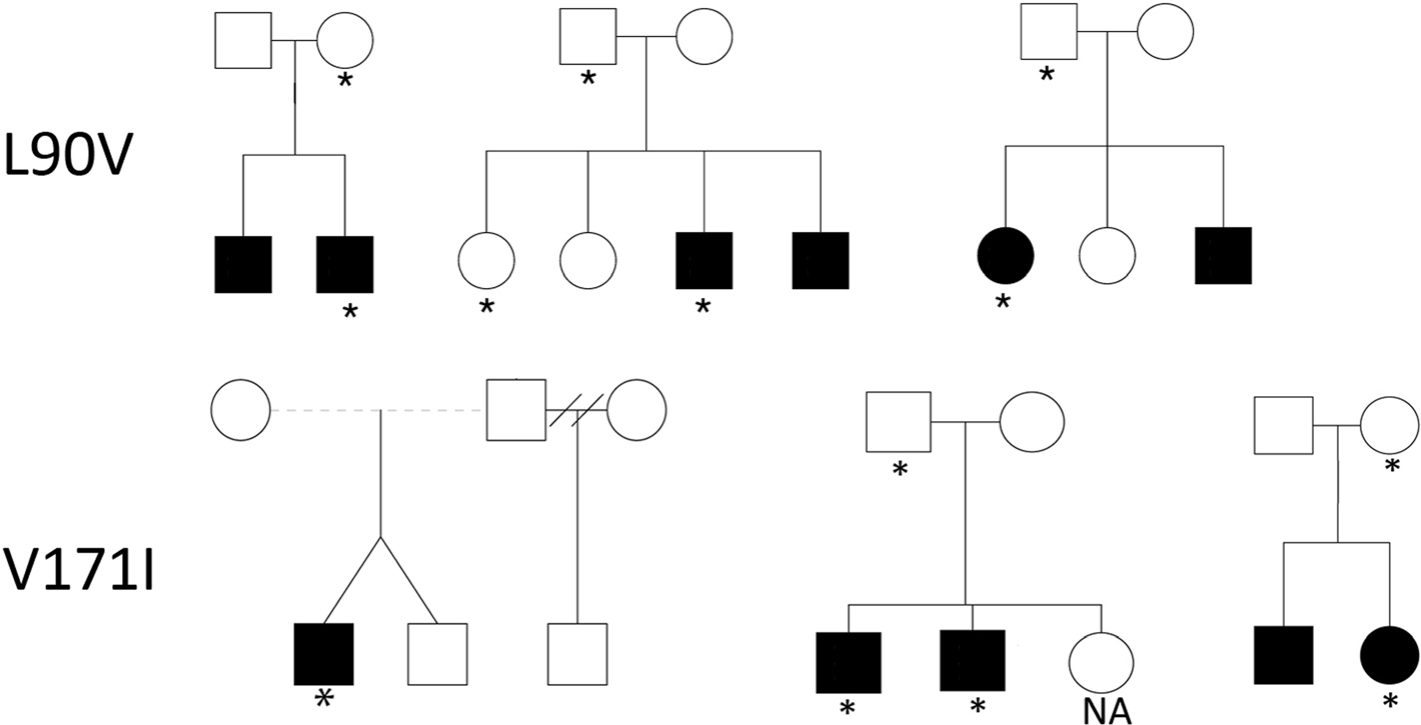
**Pedigree structure of ASD families harboring the Leu90Val or Val171Ile variants.** Subjects harboring either variant are indicated by an asterisk. Individuals for whom DNA was not available are labeled NA. Individuals with ASD are indicated by filled circles or squares, while unfilled elements reflect individuals without an ASD diagnosis.

We sought to determine whether there was evidence for an increase of rare *ADORA3* coding variants in cases compared with controls. Both Leu90Val and Val171Ile were detected only in ASD cases and not controls (3/370 case chromosomes for each variant *vs*. 0/562 control chromosomes). A case-control comparison of these individual rare alleles does not reach significance given the small number of observations (Fisher’s Exact *P*=0.064 for Leu90Val and Val171Ile each). Given the inherent limitation in power to compare rates of single rare alleles, a gene-wide discovery model suggests that a better approach is to model all ‘functional’ (that is, non-synonymous, consensus splice site, and read-through) rare variants simultaneously for case-control comparisons. Therefore, we employed the Cohort Allelic Sums Test (CAST) to test for an overall increased burden of rare (<1%) nsSNPs in *ADORA3* in ASD. We reasoned that any risk effect of higher frequency, common coding variants would be indexed by SNP and haplotype-based association studies described above. Allele counts obtained from sequence discovery in 185 cases and 305 controls were tabulated for Leu90Val, Val171Ile, and Ala195Thr, and a Fisher’s Exact test was conducted (Table 2). This analysis showed a significant burden effect (6/185 ASD individuals *vs*. 1/310 controls; *P*=0.013; OR=10.19, CI = 1.20-81.56). We note that, as described in Methods, we have low power to detect rare variants of only modest risk effect. Nevertheless, we observed a nominal increase in rare ‘functional’ variants in the discovery case sample *vs*. controls, and this prompted us to: (1) conduct functional studies of the novel variants found in ASD cases for effects on SERT; and (2) subsequently compare the putative increase of rare, ‘functional’ variants in ASD cases to data from exome sequence that became available from the NIH ARRA Autism Sequencing Consortium [50,52]. Consistent with published data on accepted risk CNVs (for example, 16p11.2, 1q21.1, 22q11.2, 22q13.3, and so on) [53-55], novel variants Leu90Val and Val171Ile did not always segregate to only (or all) affected individuals in a family, consistent with incomplete penetrance of these variants (Figure 1).

**Table 2:** **The Cohort Allelic Sums Test (CAST) on rare *ADORA3 *** **variants in discovery cohort**

**rs#**	**mRNA**	**Cases**	**Controls**
rs77883500	Leu90Val	3	0
rs139935750	Val171Ile	3	0
rs143962803	Ala195Thr	0	1
	Carriers	6	1
	Non-carriers	179	304

*P*=0.013 (OR=10.19, CI: 1.22-85.37).

**Figure 2:**
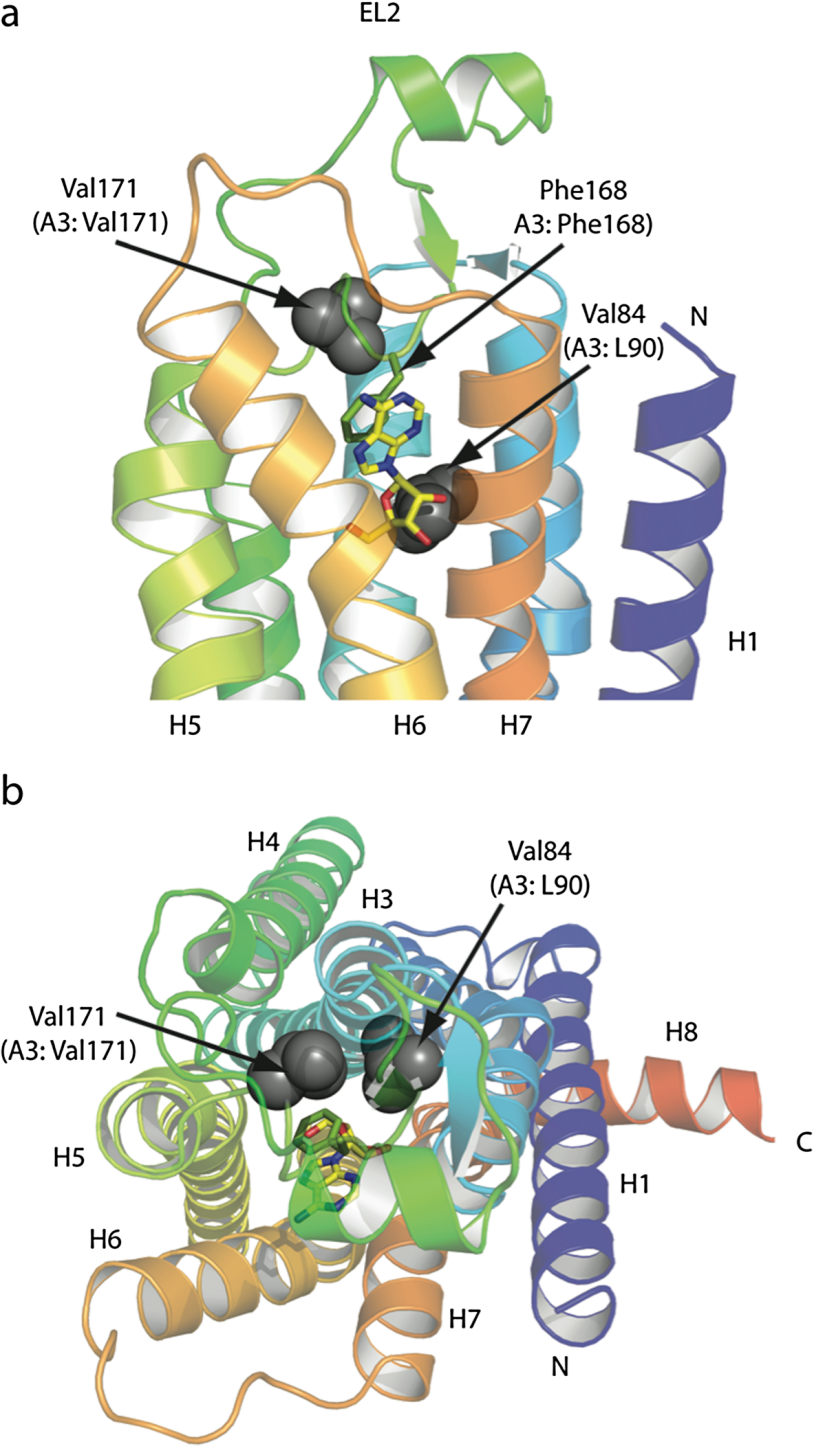
**Structure of the A2a adenosine receptor identifying corresponding positions of Leu90 and Val171 residues in the A3 receptor.** The structure of the thermostabilized A2a receptor, A2aAR-GL31 (PDB code 2YDO), is depicted as a cartoon in rainbow coloration (N-terminus in blue, C-terminus in red), viewed either **(a)** parallel to the membrane plane or **(b)** from the extracellular surface perpendicular to the membrane plane. The endogenous ligand adenosine is shown as a stick model (C, yellow, N, blue, O, red) as is the side chain for Phe168 (C, green). The transmembrane helices are labeled H1-H7, the extracellular loop 2 (EL2) is indicated and H8 is the C-terminal amphipathic helix lying parallel to the membrane plane. A3AR residues Leu90 and Val171 correspond to Val84 and Val171 in A2aAR, and they are both shown as space-filling models (grey).

**Figure 3:**
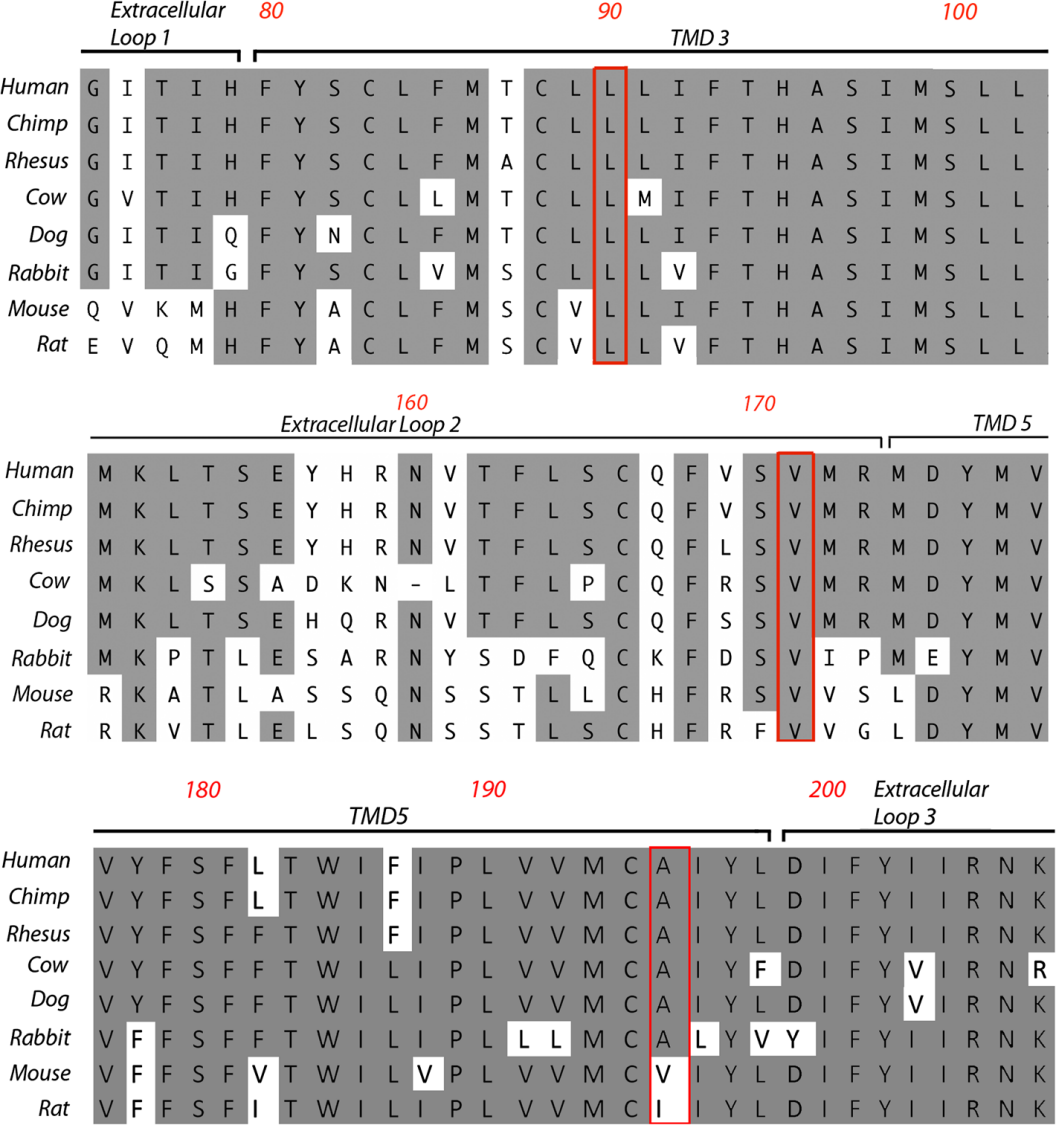
**Cross-species conservation at *ADORA3 *variant sites detected by Sanger sequencing.** Amino acid sequences of the A3AR protein encoded by *ADORA3* are aligned for the three variants and their flanking residues: Leu90Val, Val171Ile (cases), and Ala195Thr (controls).

The availability of crystal structure for a human ligand-bound adenosine A2a receptor (A2aAR) [56] provides an important source of structure-function information that can inform predictions of a potential functional impact of the Leu90Val and Ile171Leu variants. Figure 2 depicts the structure of the A2aAR, modeling the positions of residues equivalent to Leu90 and Val171 in A3AR, in relation to bound adenosine. Our model predicts that the A3AR residues Leu90 and Val171 flank the adenosine-binding site, supporting a hypothesis that one or both of these variants may affect A3AR function. Leu90 and Val171 show consistent cross-species conservation in mammals, while Ala195 is less conserved (Figure 3). Functional studies therefore focused solely on the two variants identified in cases.

### Functional analysis of Leu90Val- and Val171Ile-A3AR effects on cGMP production and SERT activity

**Figure 4:**
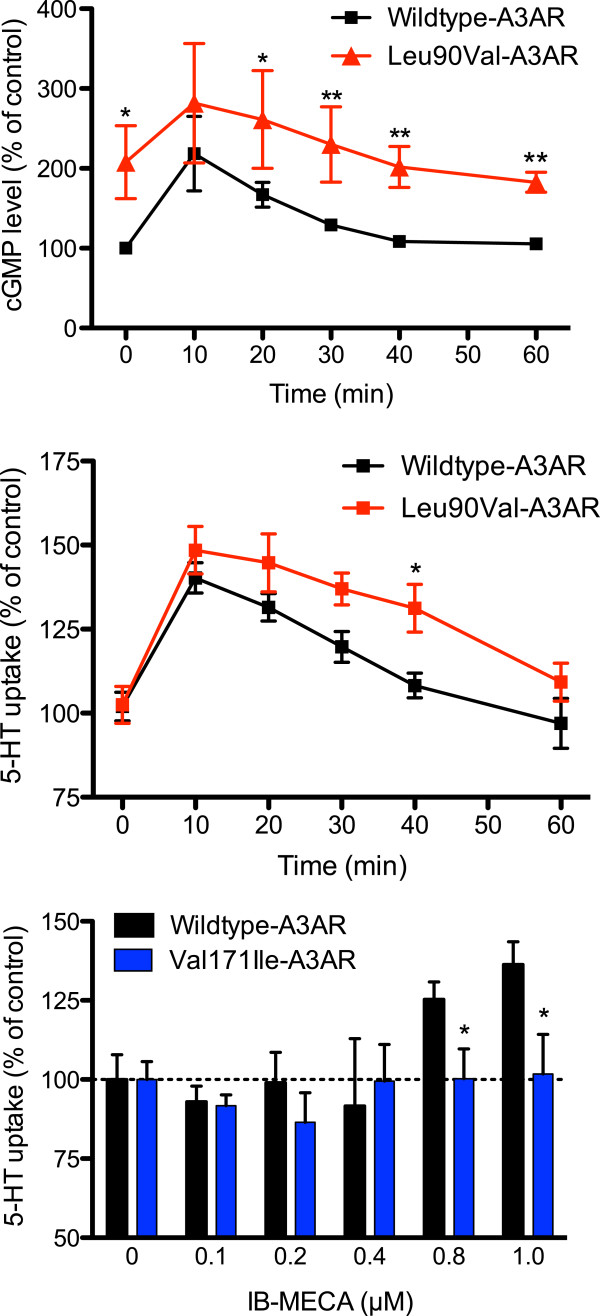
**Functional consequences of the A3AR specific agonist IB-MECA on WT-A3AR and Leu90Val-A3AR or Val171Ile-A3AR expressing cells.** CHO cells co-transfected with either WT-A3AR or Leu90Val-A3AR or Val171Ile-A3AR and SERT and were stimulated using IB-MECA (1 μM). **(A)** Levels of cGMP production from WT-A3AR/SERT and Leu90Val-A3AR/SERT co-transfections were measured prior to and after IB-MECA stimulation. Leu90Val-A3AR/SERT cells display elevated basal cGMP production that persists over the entire 60 min time course experiment, revealing enhanced overall production of cGMP (1 tailed t-test *P*=0.049; *n*=3). Each of the three independent experiments contained a minimum of three internal replicates. **(B)** 60 min time-course of IB-MECA induced 5-HT uptake as measured as percent of control (wildtype; time = 0 min). CHO cells were co-transfected with either wildtype-A3AR and SERT or Leu90Val-A3AR and SERT, and 5-HT uptake was stimulated using IB-MECA (1 μM). Cells were incubated with [^3^H]5-HT (20 nM) for the indicated period. Over the time course (0-60 min) in Leu90Val-A3AR/SERT expressing cells, 5-HT uptake is elevated compared with the wildtype A3AR counterpart (1 tailed t-test, *P*=0.039; *n*=4). Each independent experiment contained a minimum of three internal replicates. **(C)** [^3^H]5-HT accumulation in cells co-transfected with A3AR and human SERT was measured, and indicate a failure of IB-MECA treatment of Val171Ile-A3AR/SERT to induce SERT dependent 5-HT re-uptake across all tested concentrations. Compared to the peak concentrations (0.8 μM and 1.0 μM) of IB-MECA induced 5-HT uptake in WT-A3AR/SERT, IBMECA induced 5-HT uptake was significantly reduced (for example, 1 μM IB-MECA Val171Ile: 101.8% ± 12.5 *vs*. WT: 136.3% ± 7.2; two-way ANOVA *P*=0.005; *n*=3-4). Significant (*P* <0.05) findings are indicated by an asterisk (*).

To test for potential functional effects caused by the Leu90Val and Val171Ile substitutions, we engineered human A3AR cDNA expression constructs to harbor either Leu90Val or Val171Ile variants for experimentation in a heterologous transfection system using CHO cells. In prior studies, we have found that CHO cells support both the investigation of A3AR regulation of SERT as well as studies of A3AR/SERT physical association [29,30]. Western blot analysis of cell lysates using an anti-myc probe confirmed equivalent expression of myc-tagged wildtype and both variant A3AR constructs, Leu90Val [29] and Val171Ile (data not shown). We first asked whether either variant (G-protein coupled) receptors produced increased (or otherwise altered) levels of cGMP that might result in a downstream increase of SERT activity. The human A3AR and SERT constructs were co-transfected into CHO cells, and we assessed basal cGMP levels for both variants, and then following activation of receptors with the A3AR selective agonist IB-MECA. Figure 4A (at 0 min) demonstrates that CHO cells co-transfected with the Leu90Val variant display significantly elevated basal cGMP compared to wildtype A3AR co-transfected cells (L90V: 207.85% ± 45.71 *vs*. WT: 100.0% ± 0.02; *P*=0.015, *n*=3). Stimulation of the Leu90Val A3AR by IB-MECA (1 μM) revealed that the elevated cGMP production seen in the basal state persisted over the full time course and paralleled wildtype A3AR with both returning to their respective basal states after 40 min (Figure 4A and Additional file 4: Figure S1A; L90V: 163.3% ± 29.15 *vs*. WT: 100.0% ± 6.32; 1-tailed t-test *P*=0.049, *n*=3).

In contrast with Leu90Val, cells co-transfected with Val171Ile-A3AR/SERT displayed basal cGMP levels similar to those observed in wildtype A3AR co-transfected cells (V171I: 116.1% ± 16.3 *vs*. WT: 100.0% ± 9.2, *n*=3). Additionally, IB-MECA treatment in Val171Ile co-transfections failed to show any stimulation of cGMP production that was seen with wildtype A3AR. This inability of the Val171Ile receptor to be stimulated by IB-MECA appeared as a slightly diminished cGMP synthesis over the time course compared with wildtype, although this difference was not statistically significant (Additional file 4: Figure S1A; V171I: 90.14% ± 12.22 *vs*. WT: 100.0% ± 16.18; 1-tailed t-test *P*=0.303, *n*=3).

The IB-MECA induced stimulation of cGMP production in wildtype A3AR/SERT co-transfections displayed in Figure 4A paralleled the expected stimulation of SERT-mediated 5-HT uptake activity by wildtype A3AR (Figure 4B and [30]). Although the elevation in peak 5-HT uptake (that is, at 10 min) in Leu90Val A3AR/SERT co-transfections was not significantly greater than that for wildtype A3AR (Figure 4B; L90V: 148.5% ± 7.1 *vs*. WT: 140.3% ± 4.5; *n*=4), the IB-MECA-stimulated increase in 5-HT uptake activity mediated by the variant receptor persisted above that seen in cells transfected with the wildtype receptor throughout the time of our observations. Indeed, the significantly increased 5-HT uptake, seen at 40 min for Leu90Val A3AR transfected cells, the time by which wildtype A3AR-stimulated SERT activity had already returned to baseline levels (L90V: 131.3% ± 7.1 *vs*. WT: 108.3% ± 3.7; two-way ANOVA *P*=0.001, *n*=4) did not return to wildtype levels until the next time point measure, 20 min later (Figure 4B). These observations reflect an overall greater 5-HT uptake in Leu90Val-A3AR cells compared to wildtype A3AR (Additional file 4: Figure S1B; L90V: 112.2% ± 8.80 *vs*. WT: 100.0% ± 3.22; 1-tailed t-test *P*=0.020, *n*=4).

In striking contrast to the enhanced SERT activity observed with Val90 co-transfected cells, IB-MECA treatment of Ile171, compared with wildtype co-transfected cells failed to stimulate SERT activity (Figure 4C) across all agonist doses tested (for example, 1 μM IB-MECA Val171Ile: 101.8% ± 12.5 *vs*. WT: 136.3% ± 7.2; two-way ANOVA *P*=0.005; *n*=3-4).

### Replication test of an increased rate of rare functional variants in cases *vs*. controls

With support from both initial Sanger discovery experiments and subsequent functional studies that potentially ASD-related, functional variation had been identified in *ADORA3*, we sought replication of the putative increase of such variants in ASD cases *vs*. controls. We examined whole exome sequence data from 339 cases and pair-matched controls of European ancestry generated by the NIH ARRA Autism Sequencing Consortium [50] for the presence of functional variants at *ADORA3* (see methods for exclusion criteria). Nine non-synonymous and numerous synonymous variants were discovered in these pair-matched samples that were completely independent from the discovery sample. In total, nine of 12 subjects harboring non-synonymous variants were detected in cases, and the remaining three in controls (9/339 ASD individuals *vs*. 3/339 controls); although that relative increase was not statistically significant (Fisher’s Exact 1-tailed *P*=0.071; OR = 3.06, CI: 0.82-10.99; Table 3). Specifically, Leu90Val was found in one additional case and no controls, while Val171Ile was found in two additional cases but also one control. Of the remainder, four additional missense variants (Ile22Thr, Phe48Ser, Ala69Ser, and Leu294Thr) were found in one case each and no controls, and a single read-through mutation (*319Gln) predicted to add an additional 38 residues (>10% of the native protein) was found in two cases and zero controls. Two additional missense variants were detected in controls (Phe180Leu and Ala273Thr). Each of these variants passed stringent quality control thresholds and have read-depths that, based on empirical data, have an extremely high positive predictive value for being valid calls [50], although they have not been experimentally validated. Prediction of functional consequences using PolyPhen2 [45] indicated that two missense variants were forecast to be benign (Ala69Ser and Leu294Thr) and four missense to be damaging (Ile22Thr, Phe48Ser, Phe180Leu, and Ala273Thr). Combining both discovery and replication findings strengthens evidence for an increase of ‘functional’ variants in ASD, with counts of 15 of 524 cases compared with four of 644 controls harboring such variants (Fisher’s Exact 1-tailed, Bonferroni-corrected *P*=0.0025; OR = 4.72, CI: 1.56-14.30). Moreover, taking into account replication data, the enrichment of Leu90Val in ASD cases *vs*. controls becomes statistically significant (Fisher’s Exact 1-tailed *P*=0.040; 4/1,048 ASD *vs*. 0/1,288 control chromosomes). Although present in greater numbers in ASD cases, enrichment for Leu171Ile by itself did not reach significance (Fisher’s Exact 1-tailed *P*=0.068; 5/1,048 ASD *vs*. 1/1,288 controls).

**Table 3:** **The Cohort Allelic Sums Test (CAST) on rare *ADORA3 *** **variants in replication cohort**

**rs#**	**mRNA**	**Cases**	**Controls**
rs112045912	Ile22Thr	1	0
	Phe48Ser	1	0
	Ala69Ser	1	0
rs77883500	Leu90Val	1	0
rs139935750	Val171Ile	2	1
	Phe180Leu	0	1
	Ala273Thr	0	1
	Leu294Phe	1	0
rs112042574	*319Gln	2	0
	Carriers	9	3
	Non-carriers	330	336

* Indicates termination codon.

*P*=0.071 (OR=3.06, CI: 0.82-11.39).

### Exploring phenotypic and genomic characteristics of *ADORA3* variant carriers

We explored the possibility that dimensions of the ASD phenotype might stand out in affected carriers of Val90 and Ile171 or other *ADORA3* variants. We examined domain and item-level data from the ADI-R to explore the possibility that certain ASD features might be more pronounced in *ADORA3* rare variant-carriers. Such comparisons are inherently limited by a small number of carriers relative to hypothetical phenotypic effect sizes and different mechanistic effects on A3AR for a given variant. Our examination of available data showed no consistent pattern of elevated or diminished ADI-R domain scores in carriers *vs*. non-carriers (Additional file 5: Table S4). We found a similar lack of correlation upon examination of ADI-derived principal components analysis-derived scores [57]), as a complementary set of dimensional ASD traits (data not shown).

To determine whether rare *ADORA3* variant carriers also harbored other known or likely risk factors, we examined data from published work describing CNVs in respective samples [58,59]. Some inherited CNVs were identified in the six cases for which CNV data were available; for the remaining nine samples, CNV data were not available and several of these have not been subjected to array based genotyping or analysis. Most CNVs corresponded to regions also detected in controls, however, a single *de novo* duplication of approximately 211 kb (hg18: ChrX:153263157–153474401) was found in the male proband (NIMH ID: 217-14216-3470) harboring a Ala69Ser substitution in *ADORA3*.

## Discussion

Based upon our knowledge that SERT and 5-HT have a longstanding connection to ASD, that A3AR plays a key role in SERT regulation via PKG and p38 MAPK signaling pathways, and that SERT is an essential regulator of 5-HT signaling, we screened *ADORA3* for SERT-altering and ASD-associated alleles. Our experiments were premised on a specific hypothesis: functional risk variants at *ADORA3* would lead to a signaling-mediated elevation of SERT-dependent 5-HT uptake activity, phenocopying *in vitro* the elevated function seen with rare, ASD-associated SERT coding variants [17,20,23]. Regarding association of ASD with SERT variants, we note that sequencing of *SLC6A4* by another group testing unrelated ASD probands from singleton or multiplex families (and controls) but without obligate allele sharing (linkage) at 17q11.2 did not show a similar pattern of enriched coding variants, and thus association with ASD risk [60]. We believe therefore that screening of multiplex probands with allele sharing in this region was an important factor in our initial discovery of novel, functional variants, given the extreme genetic heterogeneity underlying ASD. Moreover, results from genotyping of ASD and related cohorts by Delorme and colleagues [20,21] further support ASD association initially suggested by Ozaki et al. [20,21] and reinforced by our own studies.

Consistent with results from large-scale GWA scans for common allele susceptibility effects [35,43], we found no evidence to support a main effect on ASD association attributable to common variants at *ADORA3*. The current study is limited in power to detect alleles of small effect size (for example, OR <1.3), so we cannot exclude the possibility that common *ADORA3* variants confer very small effect sizes or interact with alleles at other genes to confer risk.

Mechanistically, we anticipated that a more likely scenario was for *ADORA3* to harbor coding variants, likely rare, that might impact A3 function directly and SERT function indirectly. Numerous, recent studies focused on CNV or sequencing [44,50,58,61-64] have documented that rare variation affecting a large number of genes is collectively a major source of genetic liability in autism. Our results are consistent with *ADORA3* being one such gene that contributes to ASD liability in rare cases. Here, we present the identification of novel coding variants in *ADORA3* in a Sanger sequencing-based screen of cases and non-clinical comparison samples ethnically matched to the case sample. This screen showed a statistically significant increase in coding variants in cases *vs*. controls (*P*=0.013). Subsequent availability and analysis of exome sequence data from cases and clinically-screened controls [50] showed a greater number of ‘functional’ variants at *ADORA3* in cases compared with controls (15/524 *vs*. 4/644), but a difference that was not significant (*P*=0.07). Nevertheless, combining Sanger discovery and newly available exome sequence data strengthens evidence for association, even after Bonferroni correction for multiple comparisons (Fisher’s exact 1-tailed *P*=0.0025; OR = 4.72, CI: 1.56-14.30).

Encouraged by the initial discovery of Leu90Val and Val171Ile, functional studies focused on these two rare non-synonymous variants identified from the ASD discovery sample. To test our central SERT regulatory network hypothesis, analysis of Leu90Val and Val171Ile examined the effect of receptor stimulation to induce cGMP synthesis via G-protein coupling and on downstream SERT-dependent 5-HT uptake. IB-MECA stimulation of the Val90-encoded A3AR showed enhanced cGMP synthesis compared with wildtype A3AR under basal conditions, and enhanced cGMP levels extended over the full time course ending with a return to the elevated baseline. These results mirrored our findings that the IB-MECA induced increase in SERT-dependent 5-HT uptake activity across the experimental time course including a delayed return to baseline in co-transfections with the Leu90Val mutant A3 receptor compared with wildtype A3AR. Thus, overall 5-HT uptake is significantly increased in cells expressing the mutant A3 receptor. Increases in both cGMP and 5-HT uptake activity parallels our recent report of enhanced Leu90Val-A3AR/SERT complex formation [29], which implies (1) enhanced basal receptor-G protein coupling, (2) reduced receptor desensitization, and/or possible differences in the binding kinetics of IB-MECA to Val90-A3AR as a consequence of its location proximal to the A3AR ligand binding site upon agonist stimulation. Taken together, our results are consistent with our prior expectation, based on increased 5-HT uptake caused by rare autism-associated coding mutations in SERT [17,20,23]. In particular, the Gly56Ala SERT variant, exhibited increased catalytic activity as would be achieved through stimulation by PKG and p38 MAPK signaling, which represent the primary pathways for A3AR-mediated upregulation of SERT.

In contrast to Leu90Val, the Val171Ile variant rendered A3AR insensitive to the selective IB-MECA agonist to induce increased cGMP synthesis and a downstream increase in SERT-dependent 5-HT uptake activity. The molecular mechanism underlying this effect is not yet clear, however, we postulate that the proximity of the Val171 residue to the ligand binding pocket may prevent or hinder the ability of the adenosine analog IB-MECA to bind A3AR, resulting in a more rapid dissociation and/or less efficient (or absence of) receptor-G protein coupling. Additional experiments will be required to fully elucidate the molecular mechanisms of these two coding variants on receptor function. Although the functional impact on SERT of the Leu90Val and Val171Ile variants are in opposite directions, it is possible that both elevated and diminished capacity for regulation of SERT through A3AR pathways can impact 5-HT clearance in a manner that disrupts 5-HT’s ability to coordinate brain development [65,66] and/or adult 5-HT signaling [67,68].

We initially conceptualized dysregulation of a SERT regulatory network based not only on the molecular impact ASD-associated SERT mutations, rather within a broader context that implicates disruptions in 5-HT signaling in autism. Hyperserotonemia in 35% of ASD cases and efficacy of selective 5-HT re-uptake inhibitors (for example, fluoxetine, citalopram) and atypical antipsychotics (for example, risperidone) in ameliorating irritability and other anxiety-related problems in ASD are just two of many themes that implicate 5-HT dysregulation in ASD. We previously demonstrated a proof-of-principal for genetic variation in SERT-binding and regulatory proteins being associated with ASD. Here we refer to the common Leu33Pro variant in *ITGB3*, which has been statistically associated with both elevated 5-HT levels in blood [9] and ASD risk [69], and which causes allele-dependent effects on SERT activity and regulation [70]. We recently observed hyperserotonemia in a knock-in mouse model of the Gly56Ala SERT variant, along with p38 MAPK-dependent hyperphosphorylation of SERT, increased SERT-dependent 5-HT clearance and 5-HT receptor hypersensitivity *in vivo*, as well as social behavior impairments, repetitive behaviors, and deficits in communication [25]. Nonetheless, we recognize that statistical association of rare *ADORA3* variants (especially Leu90Val) requires further validation in larger samples. This is the case for rare variants in any specific locus implicated in ASD, and has led many investigators to emphasize the network as a better substrate to elucidate the underlying mechanisms. We recognize that the magnitude of ASD risk conferred by these variants is unknown. Indeed, the single male case harboring an Ala69Ser variant (of unknown functional effect) at *ADORA3* also possessed a *de novo* duplication of an X-linked interval including *RPL10*, a gene for which inherited and *de novo* point mutations and gene-disrupting and/or CNV deletion has been associated with ASD and ID [71]. Duplication effects here are unknown, but this variant is likely to confer risk. Nevertheless, we believe these studies will add to the growing body of data implicating specific gene/protein networks in contributing to ASD liability. Taken together our studies support the case for 5-HT and more specifically SERT regulatory pathways as one gene/protein network in which perturbations contribute to the underlying pathophysiology of ASD. Further studies within this network may provide new leads to ASD therapeutics.

There are a few limitations or caveats of the studies we present. First, while the discovery sequence sample was ethnically matched, subjects were not matched based on genome-wide genotype data. It is possible therefore, that subtle population stratification effects could lead to inflation of the observed increase in numbers of rare, ‘functional’ variants in cases *vs*. controls. Given that the case and control samples from the AASC were pair-matched based on genotype data, and the greater number of functional variants in those cases (15/524 *vs.* 4/644) was not statistically significant in a Fisher’s Exact test, we recognize that both discovery (*P*=0.0143) and combined (*P*=0.0025) evidence for gene-based association of rare, ‘functional’ variants should be interpreted with caution. Second, while multiple comparisons were conducted in functional experiments, we are reassured that the increases in cGMP production and 5-HT uptake parallel one another, supporting our conclusions regarding the ability of Leu90Val A3AR to augment SERT-dependent 5-HT uptake over time. Finally, we note that the functional experiments were conducted in CHO cells, and may not reflect the function during or after development *in vivo*. While this is certainly possible, our study of multiple variants in SERT and other proteins that influence SERT regulation (for example, ITGB3) present a consistent picture of results from *in vitro* transfection-based experiments ultimately relating very well to effects on SERT function in mouse models harboring these variants [70,72,73].

## Conclusions

Our results validate the hypothesis that the SERT regulatory network harbors rare, functional variants that impact SERT activity and regulation in ASD, and encourages further investigation of this network as a site for additional functional variation that may impact ASD risk.

## Abbreviations

5-HT: 5-hydroxytryptamine (serotonin); ADORA3: A3 subtype adenosine receptor; ANOVA: Analysis of variance; ASD: Autism spectrum disorder; CAST: Cohort allelic sums test; cGMP: Cyclic guanosine monophosphate; CHO: Chinese hamster ovary; CNV: Copy number variation/variant; FBAT: Family-based association test; GPCR: G-protein coupled receptor; IB-MECA: *N*6-(3-iodobenzyl)-*N*-methyl-5′-carbamoyladenosine; MAPK: Mitogen-activated protein kinase; A3AR; MDS: Multidimensional scaling; NOS: Nitric oxide synthase; OCD: Obsessive-compulsive disorder; PBS: Phosphate-buffered saline; PCR: Polymerase chain reaction; PKC: Protein kinase C; PKG: Protein kinase G; SERT, SLC6A4: serotonin transporter.

## Competing interests

The authors declare that they have no competing interests.

## Authors’ contributions

NGC, RDB, and JSS designed the study. NGC performed genetic studies including sequencing, genotyping, and exome replication analysis. NGC, CBZ, KML, and WAH performed *in vitro* functional assays. BLY and EKG preformed ancestry determination. CGT compared the A3AR with A2aAR and generated Figure 2. ARRA Sequencing Consortium performed exome sequencing and variant calls. NGC, RDB, and JSS prepared the manuscript. All authors read and approved the final manuscript.

## Supplementary Material

Additional file 1: Table S1 Characteristics of genotyped families with autism stratified by ancestry. A total of 958 families were genotype for common variants (MAF >5%). Ancestral background was determined using classical multidimensional scaling (MDS) using PLINK. Click here for file

Additional file 2: Table S2 Primers designed in Sanger sequencing discovery of the *ADORA3* gene loci. Click here for file

Additional file 3: Table S3 Association analysis of common variants in the *ADORA3* gene loci. Analysis was conducted using the family-based association test (FBAT) suite and genotype data generated from TaqMan allelic discrimination assays in a sample of 940 autism families. SNP ID, alleles, minor allele frequency (MAF), informative families, observed and expected transmission counts, and corresponding *P* values (empirical variance ‘-e’ option) are provided. Significant *P* values indicated evidence for distortion of allele transmission. Click here for file

Additional file 4: Figure S1 Activation of Leu90Val-A3AR expressing cells leads to prolonged cGMP production and 5-HT uptake. (A) CHO cells co-transfected with either WT-A3AR or Leu90Val-A3AR or Val171Ile-A3AR and SERT and were stimulated using IB-MECA (1 μM). Leu90Val-A3AR/SERT cells display elevated area under the curves (AUC) for cGMP production compared to wildtype-A3AR/SERT. (B) IB-MECA induced Leu90Val-A3AR activation enhances total SERT dependent 5-HT uptake compared to wildtype. Significant (*P* <0.05) findings are indicated by an asterisk (*). Click here for file

Additional file 5: Table S4 Autism phenotypic profile of rare variants in the *ADORA3* gene. Ancestry (AA: African American, EUR: European); IQ (Composite score on the Wechsler Preschool and Primary Scale of Intelligence (WPPSI) test); The Western Psychological Services (WPS) domain scores of the Autism Diagnostic Interview-Revised are provided. *: AGP ID, no NIMH ID available. Click here for file
