# Supplementary material for: Rare coding variants of the adenosine A3 receptor are increased in autism: on the trail of the serotonin transporter regulome
Source: Mol Autism. 2013 Aug 16;4:28. doi: 10.1186/2040-2392-4-28 (PMC3882891; doi:10.1186/2040-2392-4-28)
Supplement: Additional file 1: Table S1 — Characteristics of genotyped families with autism stratified by ancestry. A total of 958 families were genotype for common variants (MAF >5%). Ancestral background was determined using classical multidimensional scaling (MDS) using PLINK. [file 2040-2392-4-28-S1.doc]

**Additional file 1: Table S1: Characteristics of genotyped families with autism stratified by ancestry.** 958 families were genotype for common variants (MAF >5%). Ancestral background was determined using classical multidimensional scaling (MDS) using PLINK

|  | All | Caucasian | African | Asian | Hispanic | Other^b^ |
| --- | --- | --- | --- | --- | --- | --- |
| Families | 958 | 763 | 29 | 26 | 39 | 101 |
| Individuals | 4150 | 3311 | 116 | 134 | 171 | 418 |
| ASD diagnosis^a^ | 1649 | 1316 | 48 | 52 | 69 | 164 |
| Female | 316 | 248 | 8 | 8 | 21 | 31 |
| Male | 1333 | 1068 | 40 | 44 | 48 | 133 |
| Number of families with: |  |  | | | | |
| 1 affected | 317 | 252 | 9 | 2 | 13 | 41 |
| 2 or more affected | 641 | 511 | 20 | 24 | 26 | 60 |

^a^Indicates number of individuals who met criteria for (i) "autism" on the ADI-R "ASD" on both the ADI-R and ADOS or "autism" on the ADOS alone

^b^Represents families with two ancestry calls or families with at least one founder of undetermined or unknown ancestry
