# Supplementary material for: Rare coding variants of the adenosine A3 receptor are increased in autism: on the trail of the serotonin transporter regulome
Source: Mol Autism. 2013 Aug 16;4:28. doi: 10.1186/2040-2392-4-28 (PMC3882891; doi:10.1186/2040-2392-4-28)
Supplement: Additional file 2: Table S2 — Primers designed in Sanger sequencing discovery of the ADORA3 gene loci. [file 2040-2392-4-28-S2.doc]

**Additional file 2 Table S2: Primers designed in Sanger sequencing discovery of the *ADORA3*** gene loci.

| Primer Set | Forward | Reverse | Length | Annealing Temperature |
| --- | --- | --- | --- | --- |
| 1 | CGCCATTGTTGTTACTGCTG | CAGCAAAGATCCTTGGTCAAA | 585 | 60.2°C |
| 2 | GGCTAAGCAGGTGTGATGCT | TTCAGGGGTGTTTCAGGAAG | 469 | 60.2°C |
| 3 | TGCTCTTTCCATCTTTTTGCT | GCCAGCAAGATCCGTCTGTA | 425 | 58.3°C |
| 4 | AGACTGTCACTGCACATGGA | GCCCTCTTTCAACATCAAGG | 589 | 60.2°C |
| 5 | GCAGAAGATTGGAGAATTAAGAGA | AATCAGAGGGATGGCAGACC | 700 | 60.2°C |
| 6 | CCAACTCCATGATGAACCCTA | GGCTCCAAGTAGCAAGCAAG | 749 | 62.3°C |
