# Supplementary material for: Rare coding variants of the adenosine A3 receptor are increased in autism: on the trail of the serotonin transporter regulome
Source: Mol Autism. 2013 Aug 16;4:28. doi: 10.1186/2040-2392-4-28 (PMC3882891; doi:10.1186/2040-2392-4-28)
Supplement: Additional file 3: Table S3 — Association analysis of common variants in the ADORA3 gene loci. Analysis was conducted using the family-based association test (FBAT) suite and genotype data generated from TaqMan allelic discrimination assays in a sample of 940 autism families. SNP ID, alleles, minor allele frequency (MAF), informative families, observed and expected transmission counts, and corresponding P values (empirical variance ‘-e’ option) are provided. Significant P values indicated evidence for distortion of allele transmission. [file 2040-2392-4-28-S3.doc]

**Additional file 3 Table S3: Association analysis of common variants in the *ADORA3* gene loci.** Analysis was conducted using the family-based association test (FBAT) suite and genotype data generated from TaqMan allelic discrimination assays in a sample of 940 autism families. SNP ID, alleles, minor allele frequency (MAF), informative families, observed and expected transmission counts and corresponding p-values (empirical variance “-e” option) are provided. Significant p-values indicated evidence for distortion of allele transmission.

| SNP | Major/ minor allele | MAF^a^ | Inf fams^b^ | Major T_OBS_^c^ | Major T_EXP_^d^ | Minor T_OBS_ | Minor T_EXP_ | Z | p-value^e^ |
| --- | --- | --- | --- | --- | --- | --- | --- | --- | --- |
| rs7517018 | T/C | 0.145 | 266 | 671 | 668.2 | 283 | 285.8 | 0.247 | 0.809 |
| rs923 | A/G | 0.180 | 287 | 723 | 710.5 | 303 | 315.5 | 1.025 | 0.338 |
| rs10776728 | A/T | 0.323 | 403 | 883 | 861.2 | 535 | 556.8 | 1.453 | 0.162 |
| rs2298191 | T/C | 0.336 | 395 | 801 | 815.7 | 589 | 574.3 | 0.983 | 0.343 |

^a^MAF indicates Minor Allele Frequency

^b^Inf Fams indicates number of informative families

^c^T_OBS_ indicates transmissions observed; equivalent to the “S” statistic in FBAT

^d^T_EXP_ indicates transmissions expected; equivalent to the “E(S)” statistic in FBAT

^e^p-value corresponds to empirical variance “-e” option in FBAT
