# Supplementary material for: Rare coding variants of the adenosine A3 receptor are increased in autism: on the trail of the serotonin transporter regulome
Source: Mol Autism. 2013 Aug 16;4:28. doi: 10.1186/2040-2392-4-28 (PMC3882891; doi:10.1186/2040-2392-4-28)
Supplement: Additional file 5: Table S4 — Autism phenotypic profile of rare variants in the ADORA3 gene. Ancestry (AA: African American, EUR: European); IQ (Composite score on the Wechsler Preschool and Primary Scale of Intelligence (WPPSI) test); The Western Psychological Services (WPS) domain scores of the Autism Diagnostic Interview-Revised are provided. *: AGP ID, no NIMH ID available. [file 2040-2392-4-28-S5.doc]

**Additional file 4 Table S4: Autism phenotypic profile of rare variants in the *ADORA3* gene.**

Ancestry (AA: African American, EUR: European); IQ (Composite score on the Wechsler Preschool and Primary Scale of Intelligence (WPPSI) test); The Western Psychological Services (WPS) domain scores of the Autism Diagnostic Interview-Revised are provided. *: AGP ID, no NIMH ID available

|  | Leu90Val | | | | |  | | Val171Ile | | | | | |
| --- | --- | --- | --- | --- | --- | --- | --- | --- | --- | --- | --- | --- | --- |
| NIMH Sample ID^1^ | | 165-3551-0001 | 2103_256200^*^ | 60-1072-008 | 218-20036-1233001 |  | 165-3583-001 | | | 2220_1^*^ | 60-1001-001 | 72-0899-03 | 72-0907-302 |
|  |
| Discovery vs. Replication | | D | D | D | R |  | | | D | D | D | R | R |
| Gender | | M | M | M | M |  | | | M | M | M | M | M |
| Ancestry^2^ | | AA | EUR | AA | EUR |  | | | EUR | EUR | EUR | EUR | EUR |
| ASD diagnostic Classification^3^ | | Strict | ASD | Strict | Strict |  | | | Strict | Strict | ASD | Strict | Strict |
| ADI-R | |  |  |  |  |  | | |  |  |  |  |  |
| Age ADI-R (mo) | | 45 | 36 | 139 | 65 |  | | | 66 | 48 | 113 | 118 | 98 |
| Social domain | | 18 | 24 | 29 | 25 |  | | | 13 | 11 | 9 | 20 | 14 |
| Communication domain verbal/non verbal | | 16 (v) | 10 | 16 (v) | 10 |  | | | 15 (v) | 13 (v) | 16 (v) | 19 (v) | 9 (v) |
| Restricted, repetitive behavior domain | | 5 | 6 | 4 | 6 |  | | | 10 | 5 | 10 | 3 | 5 |
| Abnormality of Development | | 5 | 2 | 4 | 5 |  | | | 3 | 5 | 4 | 3 | 3 |

^1^Samples labeled with an asterisk (*) are not in the NIMH Repository and Autism Genome Project (AGP) IDs are provided

^2^Ancestry based on eigenvector analyses determined to be European (EUR) or African-American (AA)

^3^Autism corresponds to an AGP classification of “strict” or “narrow” autism; ASD corresponds to an AGP “spectrum” classification (see Methods)

|  | Ile22Thr | Phe48Ser | Ala69Ser | Leu294Phe | *319Gln | *319Gln |
| --- | --- | --- | --- | --- | --- | --- |
| NIMH Sample ID^1^ | 217-14276-3990 | 211-5202-3 | 217-14216-3470 | 215-13218-2403 | 74-0580-03 | 215-13003-43 |
|
| Discovery vs. Replication | R | R | R | R | R | R |
| Gender | M | F | M | M | M | M |
| Ancestry^2^ | EUR | EUR | EUR | EUR | EUR | EUR |
| ASD diagnostic Classification^3^ | Strict | Strict | Strict | Strict | Strict | Strict |
| ADI-R |  |  |  |  |  |  |
| Age ADI-R (mo) | 62 | 70 | 67 | 157 | 185 | 71 |
| Social domain | 25 | 24 | 18 | 26 | 29 | 20 |
| Communication domain verbal/non verbal | 10 (nv) | 20 (v) | 8 (nv) | 21 (v) | 16 (v) | 18 (v) |
| Restricted, repetitive behavior domain | 3 | 6 | 3 | 8 | 11 | 8 |
| Abnormality of Development | 3 | 2 | 3 | 3 | 3 | 3 |
